# Supplementary material for: Activation of NLRP3 Inflammasome by Virus-Like Particles of Human Polyomaviruses in Macrophages
Source: Front Immunol. 2022 Mar 9;13:831815. doi: 10.3389/fimmu.2022.831815 (PMC8959312; doi:10.3389/fimmu.2022.831815)
Supplement: Supplementary file 4 [file DataSheet_1.docx]

Supplementary Material

# Supplementary Figures and Tables

## Supplementary Figures

**
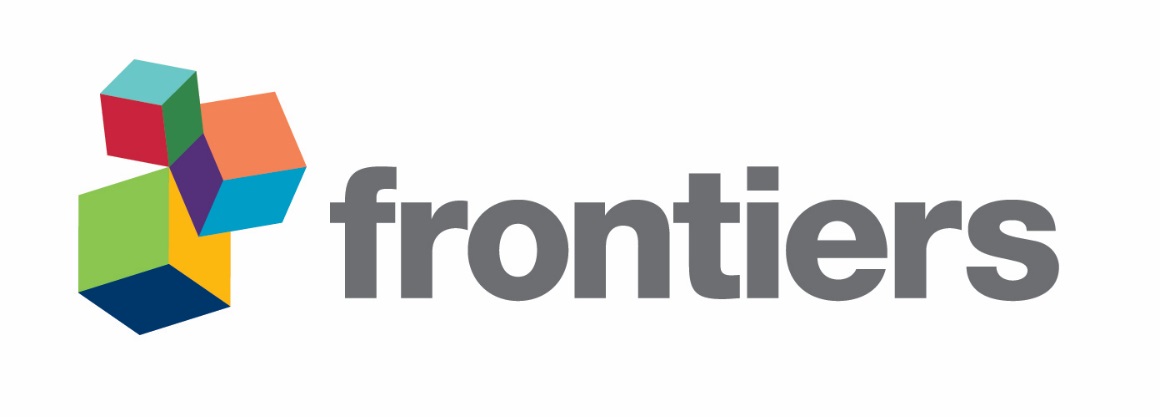
**





**Supplementary Figure 1**. **Nigericin induced cell death and IL-1β release in THP-1 macrophages.** THP-1 macrophages were treated for 24 h with nigericin (10 µM), inhibitor MCC950 (1 μM) was added 30 min before treatment. (A) PI (dead cells) and Hoechst (all cells) nuclear staining. The scale bars indicate 200 μm. “Nigericin+” – refers to Nigericin+MCC950. (B) Quantification of dead cells. (C) Cytotoxicity assessed by LDH assay. (D) IL-1β and (E) TNF-α secretion determined by ELISA. Data are represented using box plots, *p < 0.05, **p < 0.01, ***p < 0.001, ****p < 0.0001, one-way ANOVA followed by Tukey’s multiple comparison test.


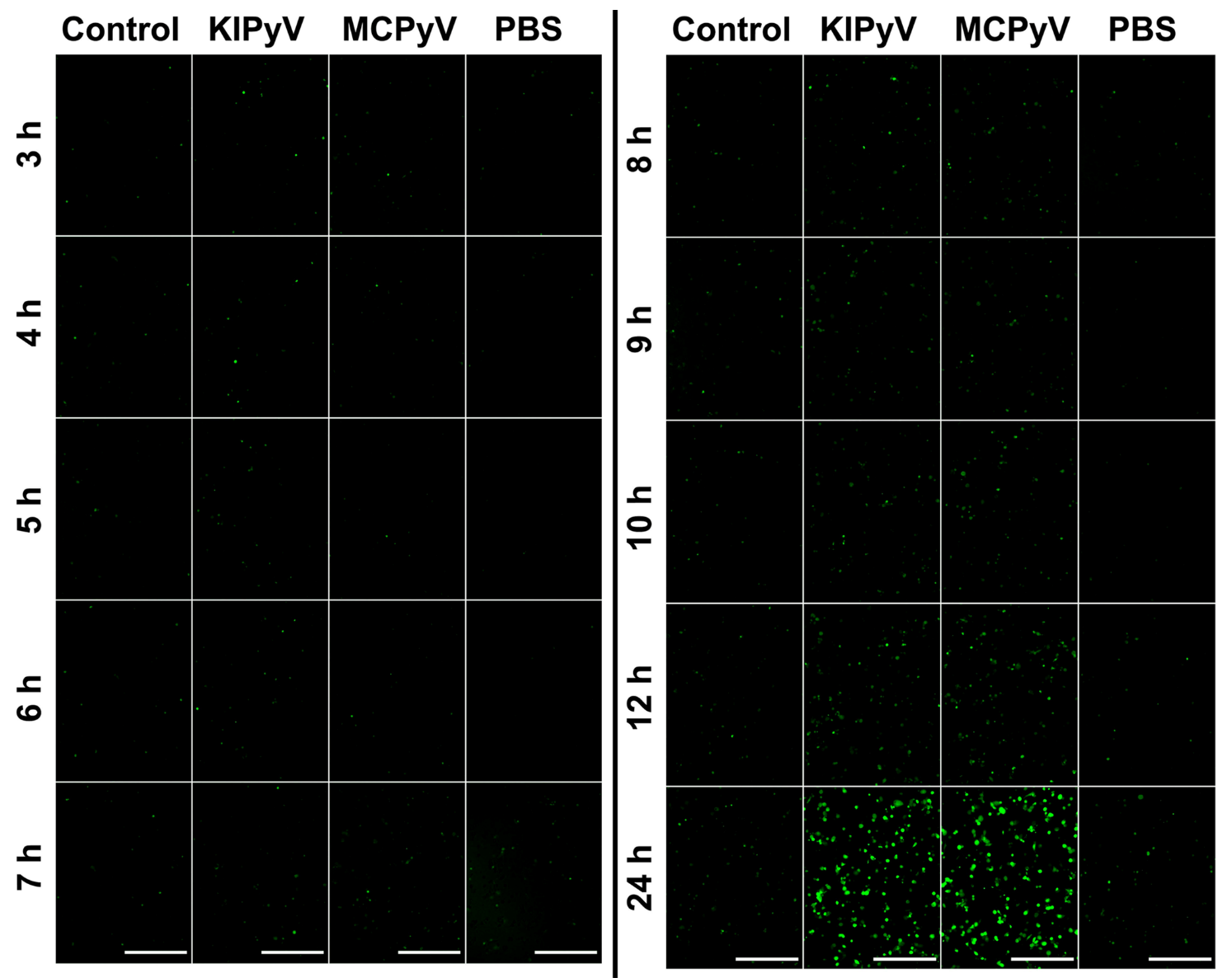


**Supplementary Figure 2A. Time lapse of ASC speck formation after PyV VLPs treatment in THP-1 macrophages.** THP-1-ASC-GFP macrophages were treated for 3-24 h with PyV-derived VLPs (20 µg/ml). Formation of ASC specks was visualised by fluorescent microscope. Representative images of one experiment are shown. The scale bars indicate 100 μm. Representative images of one experiment are shown, N = 1.


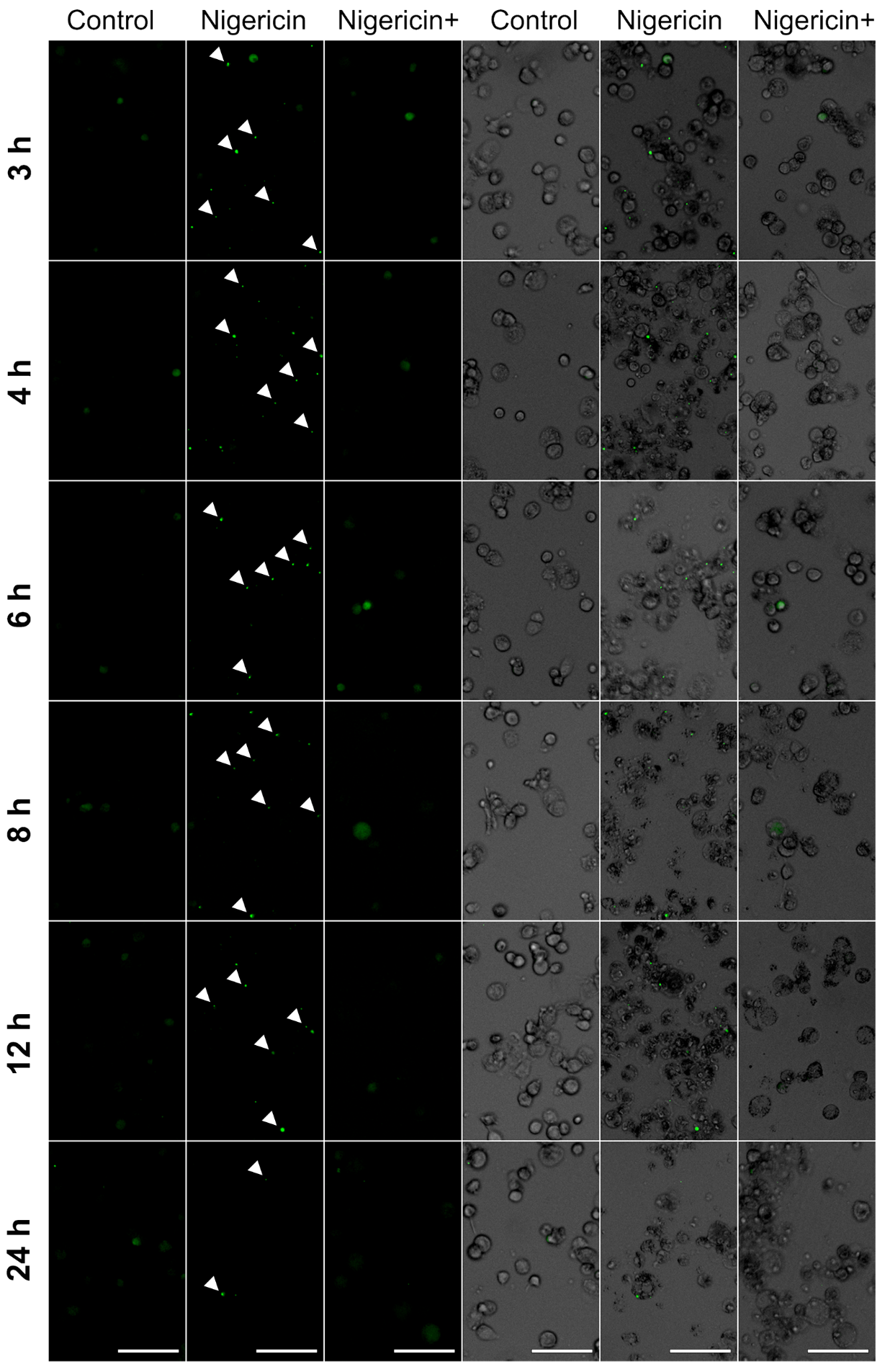


**Supplementary Figure 2B. Time lapse of ASC speck formation after nigericin treatment in THP-1 macrophages.** THP-1-ASC-GFP macrophages were treated for 3-24 h with nigericin (10 µM), inhibitor MCC950 (1 μM) was added 30 min before treatment. Formation of ASC specks was visualised by fluorescent microscope. Representative images of one experiment are shown, N = 1. Grey arrows show examples of ASC specks. “Nigericin+” – refers to Nigericin+MCC950. The scale bars indicate 50 μm.


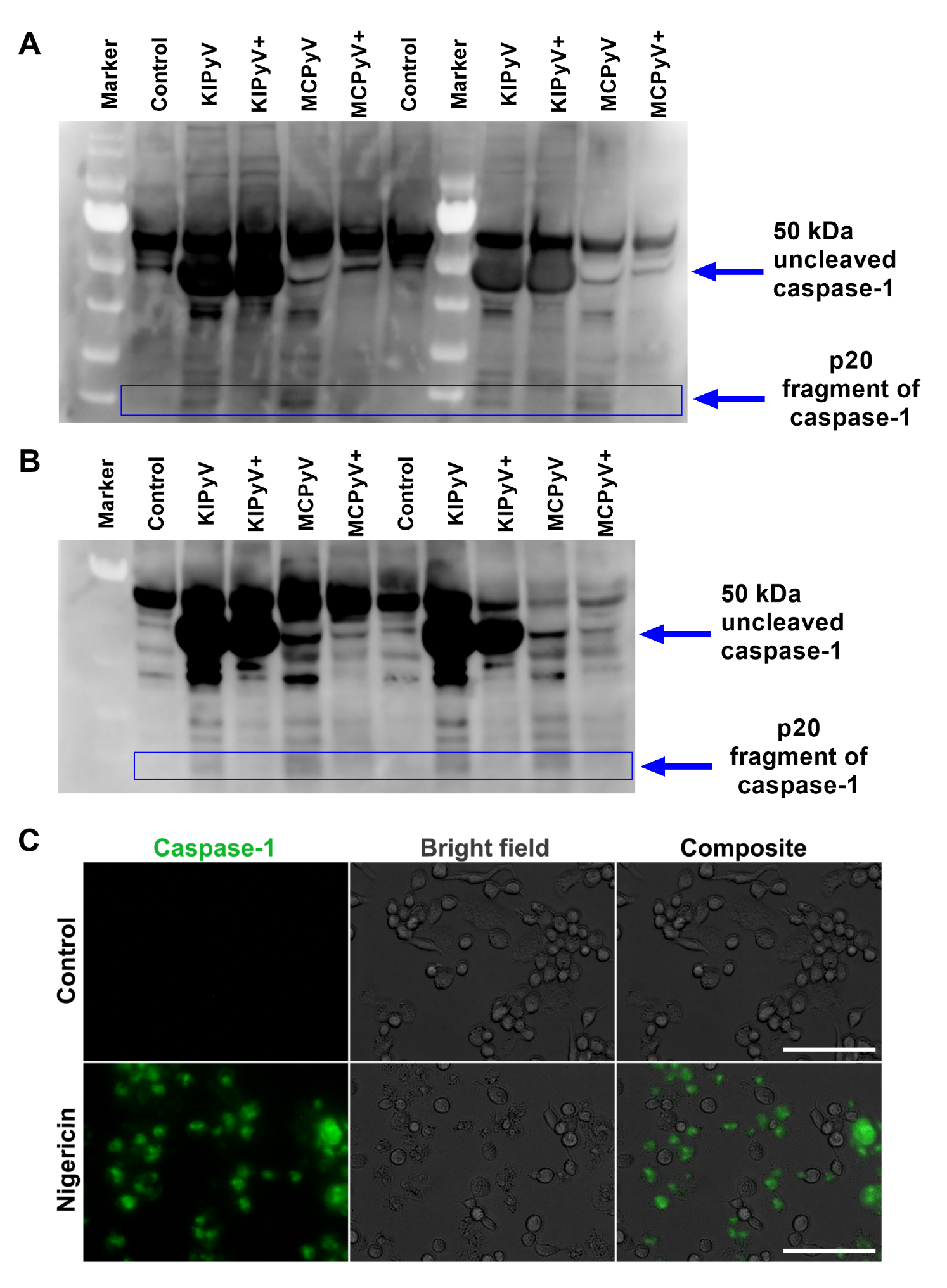


**Supplementary Figure 3. Activated caspase-1 detection after PyV VLPs and nigericin treatment in THP-1 macrophages.** Macrophages were treated for 15 h with PyV-derived VLPs (20 µg/ml), inhibitor MCC950 (1 μM) was added 30 min before treatment. (A, B) Cleaved caspase-1 was determined in cell supernatants by WB. Caspase-1: 50 kDa – pro-caspase-1; 20 kDa – cleaved caspase-1 (p20). “+” – refers to MCC950 pre-treatment. (A) and (B) show independent experiments. In one WB image duplicates of one experiment are shown. Blue rectangles show areas selected for quantification (see Figure 4B). (C) Macrophages were treated for 1 h with with nigericin (10 µM). Representative images of the activated caspase-1 staining by FLICA (green) reagent. The images were taken using 40× objective. The scale bars indicate 100 μm. N = 1.


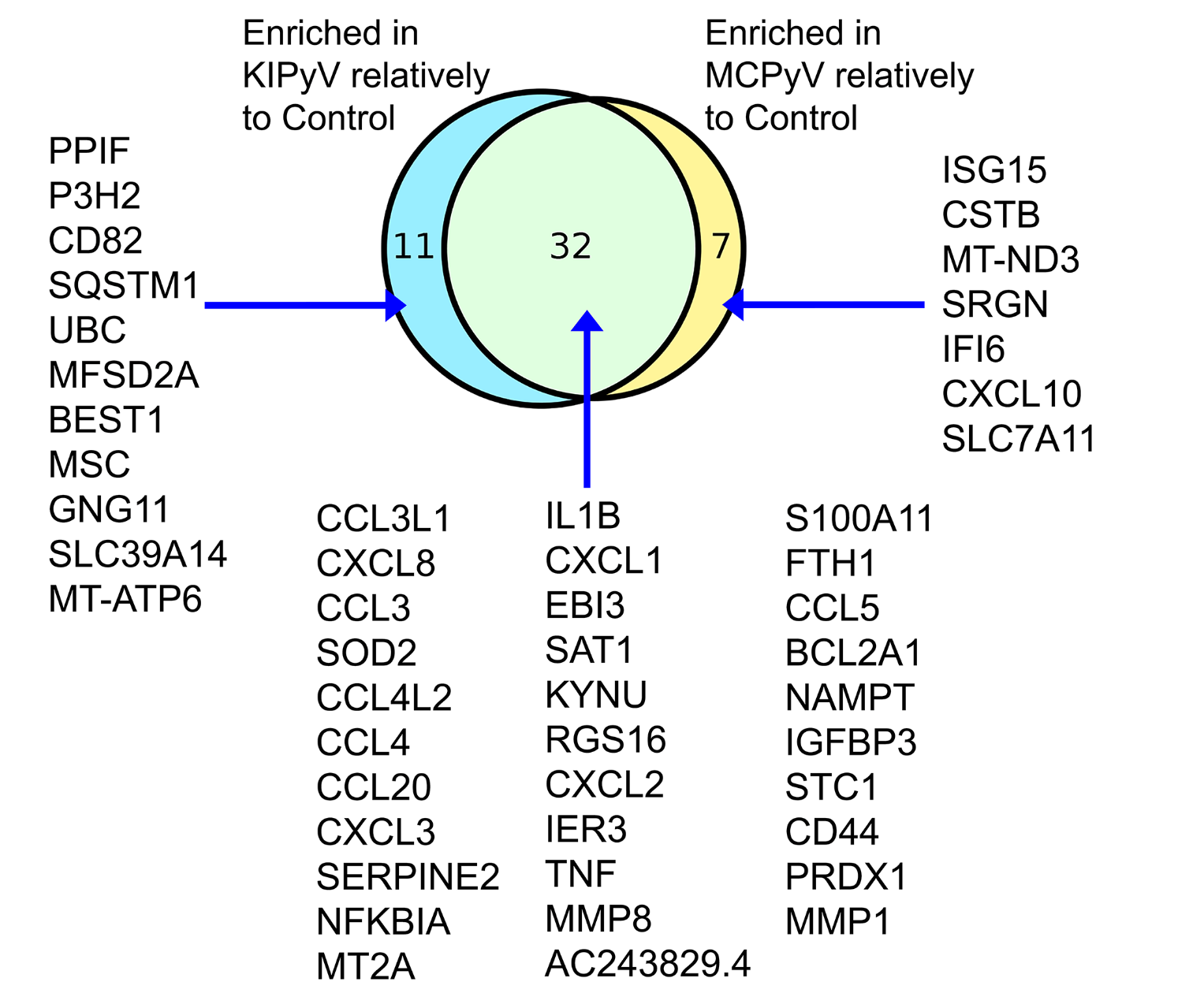


**Supplementary Figure 4. Bulk-like scRNAseq data analysis.** Volcano plot showing the result of a bulk-like comparison of MCPyV vs KIPyV VLPs. Differentially expressed genes (DGEs) were defined as having an absolute fold-change >1.5 and FDR < 0.05 (Mann-Whitney U test).


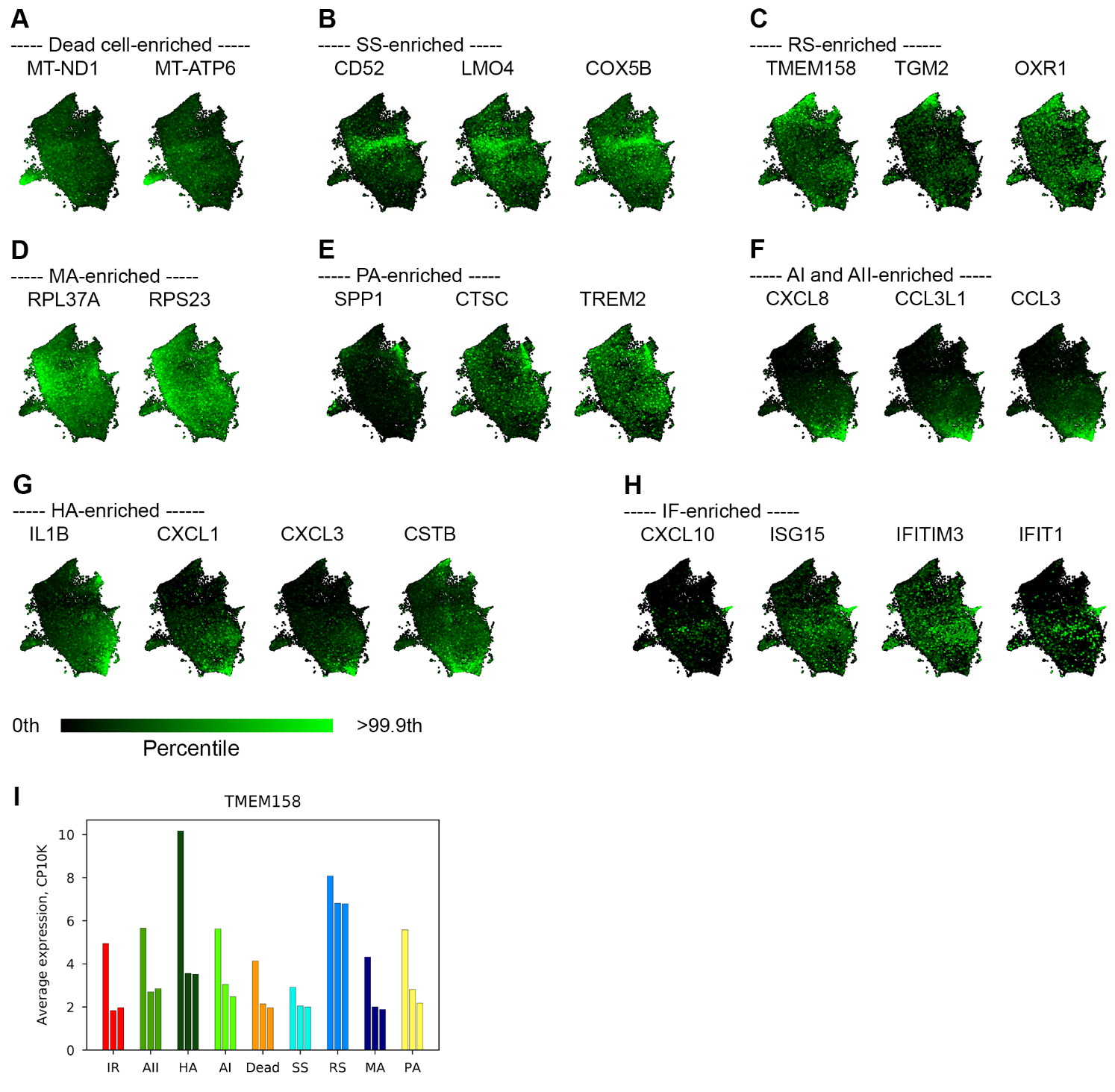


**Supplementary Figure 5**. **UMAP plots combining all conditions coloured by expression of selected genes**. (A-H) Plots of enriched genes in separate populations are shown: (A) dead cells, (B) SS population, (C) RS population, (D) MA population, (E) PA population, (F) HA population, (G) AI and AII populations, and (H) IF population. (I) Bar chart of average *TMEM158* expression in individual populations and conditions. Condition order left-to-right: Control, KIPyV VLPs, MCPyV VLPs.

**
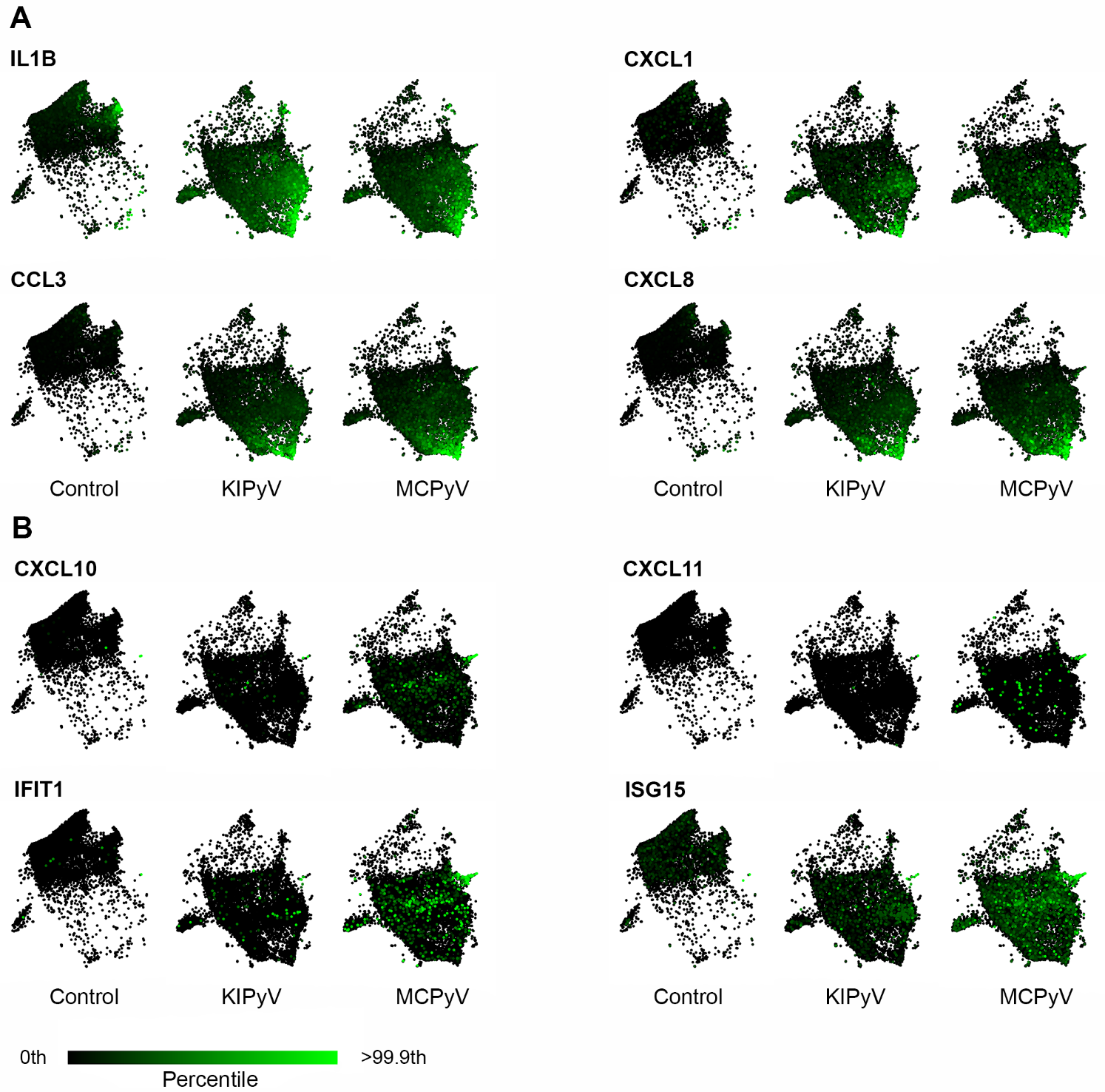
**

**Supplementary Figure 6**. **Examples of genes enriched in different treatments – control, KIPyV and MCPyV.** (A, B) UMAP plots colored by expression of selected genes in each condition separately. For a given gene, the 3 plots are saturated at the same absolute value to allow comparison. *IL1B*, *CXCL1*, *CCL3*, and *CXCL8* are examples of genes upregulated in both KIPyV and MCPyV conditions relatively to the control (A). *CXCL10*, *CXCL11*, *IFIT1*, and *ISG15* are expressed in the MCPyV-specific IR population (B).

## Supplementary Tables

**Supplementary Material Table Legends**

Ssupplementary material tables uploaded as a separate files.

**Table S1. Bulk-like scRNAseq data differential gene expression analysis results**

**Table S2. GO gene set enrichment analysis results for genes commonly enriched in VLP treated samples**

**Table S3. Data underlying Figure 9E**
